# Supplementary material for: Realization of nearly dispersionless bands with strong orbital anisotropy from destructive interference in twisted bilayer MoS2
Source: Nat Commun. 2021 Sep 24;12:5644. doi: 10.1038/s41467-021-25922-8 (PMC8463715; doi:10.1038/s41467-021-25922-8)
Supplement: Supplementary file 1 — Supplementary Information [file 41467_2021_25922_MOESM1_ESM.pdf]

Supplementary Information for Realization of nearly dispersionless bands with strong orbital anisotropy from destructive interference in twisted bilayer MoS_2

(Dated: August 28, 2021)

SUPPLEMENTARY NOTE 1: ANALYSIS AT HALF-FILLING

We start from the effective tight-binding Hamiltonian for the second-highest-energy Moiré bands for twisted bilayer MoS_2 given in the main text keeping only nearest-neighbor terms. To derive an effective spin Hamiltonian at half-filling, we start from the local triplet states

$$|-\rangle = \hat{c}^\dagger_{p_x,\downarrow}\hat{c}^\dagger_{p_y,\downarrow}|\mathrm{vac}\rangle \tag{1}$$

$$|0\rangle = \frac{1}{\sqrt{2}}\left[\hat{c}^\dagger_{p_x,\downarrow}\hat{c}^\dagger_{p_y,\uparrow} + \hat{c}^\dagger_{p_x,\uparrow}\hat{c}^\dagger_{p_y,\downarrow}\right]|\mathrm{vac}\rangle \tag{2}$$

$$|+\rangle = \hat{c}^\dagger_{p_x,\uparrow}\hat{c}^\dagger_{p_y,\uparrow}|\mathrm{vac}\rangle \tag{3}$$

that define a spin $S = 1$, with $|\mathrm{vac}\rangle$ denoting the vacuum state with no electrons. Performing a strong coupling expansion to fourth order in hopping t_σ, t_π, one obtains

$$\hat{H}_{\mathrm{eff}} = J_{\mathrm{ex}}\sum_{\langle ij\rangle}\mathbf{S}_i\cdot\mathbf{S}_j + K_{\mathrm{ex}}\sum_{\langle ij\rangle}\left(\mathbf{S}_i\cdot\mathbf{S}_j\right)^2 \tag{4}$$

where

$$J_{\mathrm{ex}} = \frac{t_\sigma^2 + t_\pi^2}{U+J} + \frac{U\left(t_\pi^2 - t_\sigma^2\right)^2 - J\left(t_\pi^4 + 6t_\pi^2 t_\sigma^2 + t_\sigma^4\right)}{J(J+U)^3} \tag{5}$$

and

$$K_{\mathrm{ex}} = \frac{t_\pi^4(J-U)(7J+3U) + 16t_\pi^2 t_\sigma^2 U^2 + t_\sigma^4(J-U)(7J+3U)}{4J(J-U)(J+U)^3} \tag{6}$$

Supplementary Fig. 1 depicts the values of the two exchange couplings as a function of Hund's exchange.

The strong anisotropy of the $\mathrm{p_x}$, $\mathrm{p_y}$ model entails that bilinear and biquadratic exchanges have the same sign, instead of opposite one like in the isotropic case. This can in principle lead to interesting phases driven by quantum fluctuations [1], however, for parameters of $J \gg t_\sigma, t_\pi$ where our strong coupling expansion is valid, K_{ex} is negliable and a classical anti-ferromagnet is found.

[1] H. H. Zhao, C. Xu, Q. N. Chen, Z. C. Wei, M. P. Qin, G. M. Zhang, and T. Xiang, Phys. Rev. B **85**, 134416 (2012).

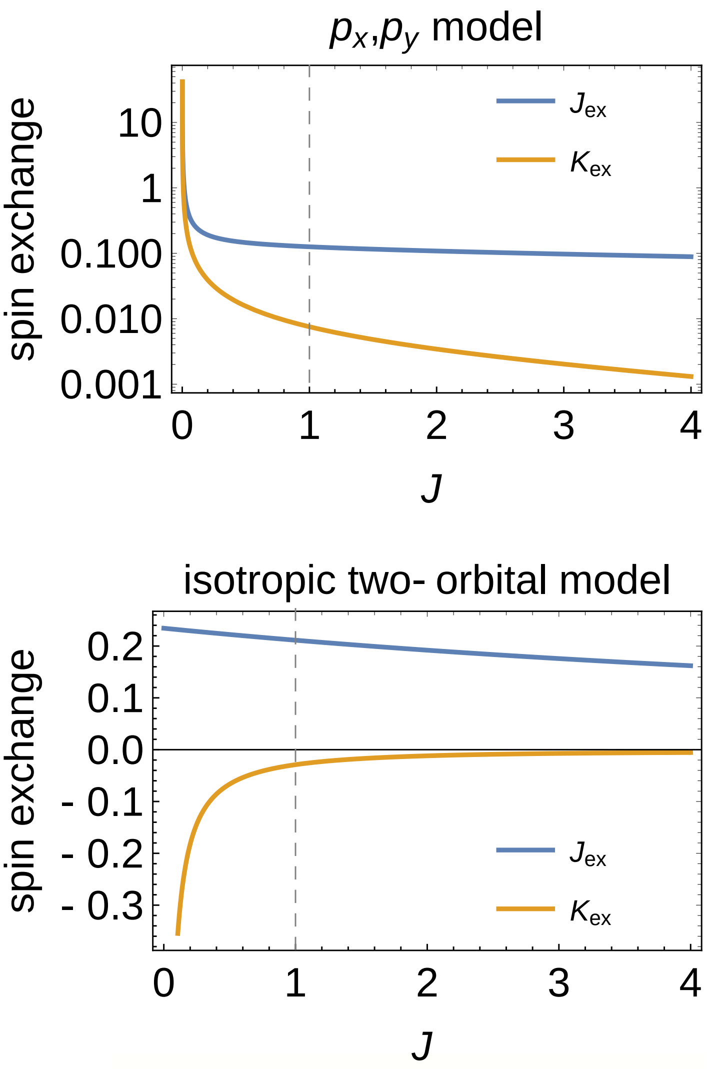

Supplementary Figure 1. **Comparison of the spin exchange couplings for the $\mathbf{p_x}$-$\mathbf{p_y}$ model and an isotropic two-orbital model.** Bilinear and biquadratic exchange couplings for $U = 8$, $t_\sigma = 1$, $t_\pi = 0.25$ as a function of Hund's exchange, for the p_x-p_y model (upper panel) and an isotropic two-orbital model (lower panel). The strong anisotropy of the p_x-p_y model entails that bilinear and biquadratic exchanges have the same sign.

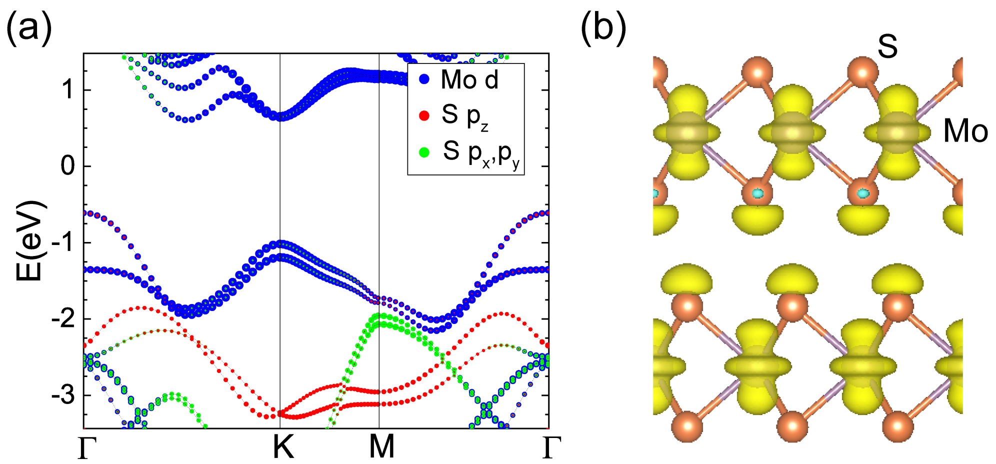

Supplementary Figure 2. **Orbital characterization of bilayer MoS_2.** (a) Orbitally projected band structure of prinstine bilayer MoS_2. The size of the dots is proportional to the projection of the wavefunction of the states to each atomic orbitals. It can be seen that the states at the K point close to the top of the valence bands are dominated by Mo d orbitals while those at the Γ point are contributed by both Mo d and S p_z orbitals. (b) Charge density distribution of the state at the Γ point at the top of the valence bands, showing both Mo d and S p_z characters.

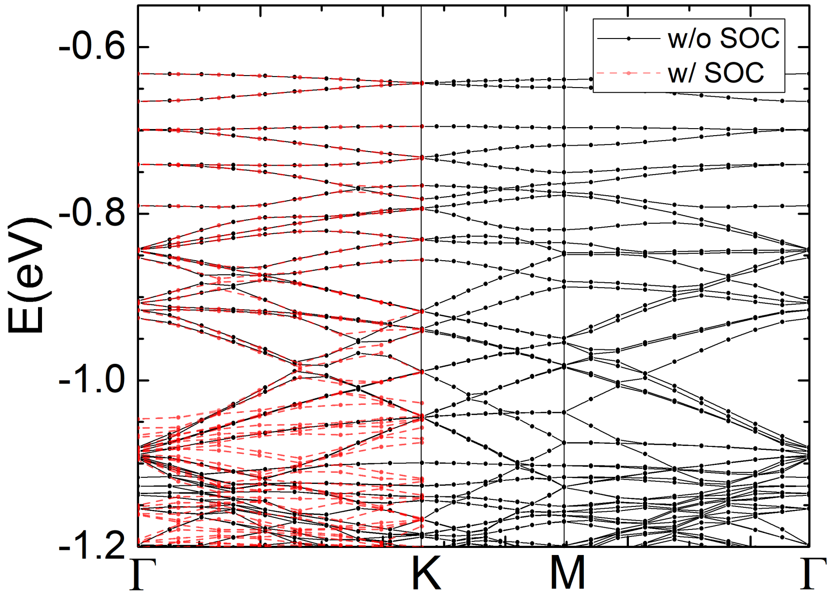

Supplementary Figure 3. **Band structures of twisted bilayer MoS_2 at 3.15 degrees with (black solid lines) and without (red dash lines) spin-orbit coupling (SOC) from Density Functional Theory (DFT) calculations.** The band structures calculated with and without SOC are identical above -1.0 eV (about 0.4 eV below the Fermi level). Therefore, when discussing the low-energy flat bands, we can ignore SOC effects.

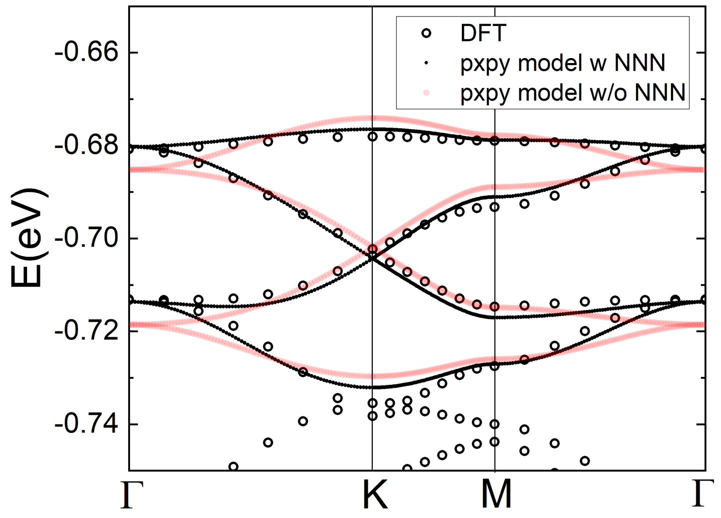

Supplementary Figure 4. **Comparison between the model fittings with and without the next nearest neighbor (NNN) hopping terms.** The DFT band structure for twisted bilayer MoS_2 at 3.15 degrees is indicated with open circles. The px-py model fitting with (without) the next nearest neighbor hopping terms is indicated with black (red) dots.

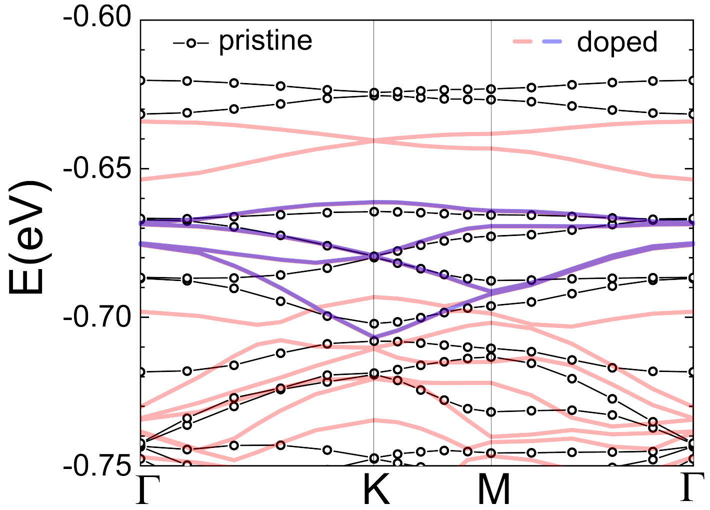

Supplementary Figure 5. **Comparison between the band structures with (black lines with open circle markers) and without doping (red and purple solid lines) for twisted bilayer MoS_2 at 2.25 degrees from DFT calculations.** For the band structure with doping, 2 electrons per supercell are removed from the system and the first two valence bands are empty. We aligned the p-like flat bands of the pristine system and the hole doped system in the figure for comparison. The px-py flat bands for the doped system are highlighted in purple. By doing so, we shifted down the Fermi level of the doped system by 0.652 eV. As shown by the comparison, the doping has three effects: (a)reduces the energy separations between the flat bands (from 32.8 meV to 7.7 meV); (b)slightly increases the band width (from 37.7 meV to 45.5 meV); (c) adds a small correction to the lower branch of the px-py flat bands. Such effects are likely to result from the reduction of the moiré potential due to the additional electrostatic potential introduced by the doping.
